# Study of the Clinical Profiling and Assessment of Poisoning Cases in a Tertiary Care Hospital

**DOI:** 10.7759/cureus.66934

**Published:** 2024-08-15

**Authors:** Abhishek Patil, Ameya A Kasture, Prasad Pathak, Shweta Patil, Sushant S Chavan

**Affiliations:** 1 Medicine, Shri Bhausaheb Hire Government Medical College and Hospital, Dhule, IND; 2 Community Medicine, Shri Bhausaheb Hire Government Medical College and Hospital, Dhule, IND

**Keywords:** india, specific treatment, supportive treatment, decontamination, tertiary care hospital, snake bites, organophosphates, pesticides, poisoning

## Abstract

Introduction

Accidental and intentional poisoning is a major cause of morbidity and mortality. Pesticide poisoning is particularly common in India, where a large percentage of the population works in agriculture. This study aims to evaluate admission profiles, management trends, and outcome status among poisoning cases in a tertiary care hospital.

Methodology

A prospective observational study was carried out from May to July 2022 in the medicine ward of a tertiary care hospital, which is associated with a government medical college. Demographic characteristics, history of poisoning, clinical presentation at the time of admission, and intervention for treatment were recorded once the patient was diagnosed with poisoning or when there was a suspicion. Data regarding outcomes was also collected from this section. The appropriateness of the decontamination, support, and specific treatments was assessed. The collected data was subjected to descriptive statistical analysis.

Results

The most common agent was pesticides, involved in 44 (43.56%) cases out of a total of 101 poisoning cases, with the predominant subtype being organophosphate. Bites accounted for 18 (17.82%) cases, mainly snake bites. Household products were responsible for eight (7.92%) cases, and medicinal products for four (3.96%) cases. Decontamination, when indicated, was properly applied in 98 (97.02%) cases; supportive treatments were administered in 95 (94.05%) cases; and specific detoxifying measures were taken in 59 (58.41%) cases. A majority of the patients (60, or 59.41%) reached the hospital within three hours of poisoning, which dramatically reduced morbidity and mortality.

Conclusion

In summary, the study indicates that pesticide poisoning is prevalent in rural India, and, as such, there is an urgent need for appropriate regulation of agrochemicals and behavioural education to protect farmers. On average, the appropriateness of decontamination and supportive treatments was high (i.e., >85%), reflecting adequate initial responses. In contrast, the low level of appropriateness for specific treatments highlights gaps regarding institutional medical protocols and training. There is a need to educate the public about timely medical intervention, which can help in decreasing the mortality and morbidity associated with cases of poisoning.

## Introduction

Poisoning continues to be an important health problem in India and a major cause of both mortality and morbidity [1]. The issue is more pronounced for a significant proportion of the Indian workforce, such as agricultural workers, who are exposed to higher levels of pesticide poisoning due to unregulated use. Due to poor regulation of pesticides and the ready availability of toxic chemicals, the incidence rate is rising compared with other forms of poisoning [2]. Moreover, in India, poisoning is the most common method of suicide [3]. This leads to a self-harm mortality rate of approximately 30% to 40%, and this report clearly suggests the need for a comprehensive mental health intervention and strict regulatory policies [4].

Management of poisoning cases is difficult, especially when specific antidotes are not available. There is an increasing need for an inclusive clinical managerial protocol that could prevent high morbidity and mortality due to poisoning [5]. This includes identification at a later stage, cancer sign management, and access to treatments available for improving patient outcomes [6].

The current study was thus designed to characterize the clinical profiles and outcomes of poisoning cases in a tertiary care setting, to understand them more closely, which is indeed expected in India. The findings may be vital to public health policymakers, as well as healthcare providers and other professionals, who will use them to develop and design effective interventions that can help reduce the number of deaths related to poisoning, leading to better patient outcomes.

## Materials and methods

From May to July 2022, a prospective observational study was conducted in the medicine ward of a government medical college, which is a tertiary care hospital, after obtaining ethical clearance from the Institutional Ethics Committee (ethical number EC/NEW/INST/2020/958). Informed written consent, either from the patients themselves or from their parents or carers, was obtained before data collection. This study involved all patients admitted with established or suspected poisoning during the said period, except those who could not give informed consent. Data were collected by means of structured interviews with patients and carers, and by reviewing medical case files. The information gathered included age, gender, socio-economic status (SES), educational level attained, marital status, and occupation, among other things.

The inclusion criteria for this research comprised patients admitted with confirmed or suspected cases of poisoning during the study period, and those able to give informed consent, whereas the exclusion criteria involved any patient who could not provide informed consent, as well as those whose poisoning occurred outside the study duration. A detailed history was taken to record the type of poison used, the route through which it entered the body, the intentional or accidental nature of such an act, and the time elapsed since exposure, among other things. Clinical manifestations were documented too, which covered vital signs at admission, including reported symptoms, their severity, and any existing comorbidities, if there were any. We systematically extracted details about the treatment provided, such as interventions done while still under admission, supportive care given, and antidotes administered, from medical records, storing them properly to ensure nothing gets lost along the way until we finally achieve success. Outcomes included recovery without complications, recovery with complications, and death.

Descriptive statistics were employed in analysing the types and frequencies of different poisons used, demographic characteristics, clinical presentations displayed by patients, and their outcomes eventually recorded at the discharge point. Types of poisoning agents were categorized based on specific features exhibited by them, including the time taken before reaching the hospital after exposure, and certain treatments received, among others. The study's results have provided valuable insights into the clinical profiles and outcomes of the patients involved, thereby facilitating the development of future management strategies for similar cases.

## Results

The demographic details of the victims were broken down by age group: 11-20 years had 14 cases (five females and nine males), 21-30 years had 24 cases (five females and 19 males), 31-40 years had 20 cases (19 males and one female), 41-50 years had 12 cases (11 males and one female), 51-60 years had eight cases (seven males and one female, or 7.9%), and 61-70 years had six cases (six males and zero females, or 5.9%). The majority of victims belonged to the age group of 21-30 years; it is a known fact that individuals in this age group are more susceptible to these issues due to work pressure, marriage, family disputes, and other life-settling factors.

The male population is at 87.2%, compared to females at 12.8%. This may be because males are more often exposed to stress and strain in day-to-day life, as well as occupational hazards, than females. The study of cases revealed that the urban population accounted for 13.8% of the total population, while the rural population constituted 86.2%. The distribution of poisoning cases across different age groups, and rural and urban settings, reveals interesting patterns. In rural areas, the highest number of cases occurred in the 21-30 age group, followed by the 31-40 age group. This could be attributed to the agricultural nature of rural areas, where pesticide poisoning is more prevalent due to occupational exposure. In urban areas, the 21-30 age group also had the highest number of cases, but the overall numbers were lower compared to rural areas, which might reflect differences in occupational hazards and lifestyle factors (Table 1).

**Table 1:** Geographic distribution of poisoning cases

Category	Total cases, n (%)	Males, n (%)	Females, n (%)	Urban, n (%)	Rural, n (%)
Age group					
11-20	14 (13.9%)	9 (8.9%)	5 (5.0%)	2 (2.0%)	12 (11.9%)
21-30	24 (23.8%)	19 (18.8%)	5 (5.0%)	4 (4.0%)	20 (19.8%)
31-40	20 (19.8%)	19 (18.8%)	1 (1.0%)	3 (3.0%)	17 (16.8%)
41-50	12 (11.9%)	11 (10.9%)	1 (1.0%)	2 (2.0%)	10 (9.9%)
51-60	15 (14.9%)	14 (13.9%)	1 (1.0%)	6 (6.0%)	9 (9.1%)
61-70	16 (15.8%)	16 (16.6%)	0 (0%)	5 (5.0%)	11 (11.1%)
Overall	101 (100%)	88 (87.2%)	13 (12.8%)	22 (21.78%)	79 (78.2%)

Interestingly, the 11-20 age group had a significant number of cases in both rural and urban areas, indicating a vulnerability among adolescents and young adults. This age group is known to be at higher risk for intentional poisoning, highlighting the importance of targeted interventions and mental health support for this demographic. The overall distribution shows a higher prevalence of poisoning cases in rural areas, which is consistent with the higher agricultural activity and potential pesticide exposures. However, urban areas also face significant challenges, particularly in addressing intentional poisoning cases among younger age groups. These findings underscore the need for region-specific strategies to prevent and manage poisoning cases, taking into account the demographic and environmental factors influencing poisoning patterns.

In our study, we observed a diverse range of poisonous substances responsible for the cases admitted. The most prevalent category was organophosphates (OPCs), which accounted for a significant portion of pesticide poisonings. Additionally, celphos (aluminium phosphide), a common fumigant used in agriculture, was identified as a frequent agent in poisoning cases. Barbiturates, often associated with overdose incidents, were also recorded. Other specific medicines taken as poisonous substances included common over-the-counter medications, such as paracetamol and ibuprofen, as well as prescription medications like antidepressants (e.g., amitriptyline) and benzodiazepines (e.g., diazepam).

The data show that the highest percentages of appropriate specific treatments were observed in cases involving bites (77.78%) and household products (75.00%), followed by medicines (75.00%) and pesticides (59.09%). In contrast, miscellaneous poisoning cases had the lowest percentage of appropriate treatments (37.04%). Overall, 58.41% of all patients received appropriate specific treatment, while 41.58% did not. A Chi-square test was conducted to assess the statistical significance of these associations, yielding a Chi-square value of 9.22 and a p-value of 0.056. Since the p-value is slightly above the conventional threshold of 0.05, we fail to reject the null hypothesis, indicating that there is no statistically significant association between the type of poisoning agent and the appropriateness of specific treatment. This suggests that the appropriateness of specific treatments does not significantly vary with the type of poisoning agent in this study. Further research with a larger sample size and across multiple centres may be necessary to explore other factors influencing treatment outcomes (Table 2).

**Table 2:** Details on distribution of type of poisoning (substance) and number of victims belonging to each poison

Type of poisoning agent	Appropriate specific treatment (yes)	Percentage (%)	Appropriate specific treatment (no)	Percentage (%)	Total	Chi-square test	p-value
Pesticides	26	59.09%	18	40.91%	44	9.22	0.056
Bites	14	77.78%	4	22.22%	18		
Medicines	3	75.00%	1	25.00%	4		
Household products	6	75.00%	2	25.00%	8		
Miscellaneous	10	37.04%	17	62.96%	27		
Total	59	58.41%	42	41.58%	101		

The study analysed 101 poisoning cases based on the time interval between poison intake and hospital arrival. Most cases (60) arrived within three hours, allowing for prompt treatment. Twenty cases arrived between three and six hours, and 19 arrived between six and 12 hours, potentially complicating treatment. Two cases had undetermined intervals. Early recognition of symptoms and prompt medical attention are crucial in managing poisoning cases effectively (Table 3).

**Table 3:** Comparison of the interval between the intake of poison and the time taken to arrive at the hospital for treatment

Interval between intake and arrival at the hospital	Number of poison victims (n)	Percentage (%)
<3 hours	60	59.4%
3-6 hours	20	19.8%
6-12 hours	19	18.8%
Undetermined	2	2.0%

The treatment assessment of 101 poisoning cases included the evaluation of decontamination, supportive, and specific treatments. Decontamination methods were deemed appropriate in 98 cases (97.02%) and inappropriate in three cases (2.98%). Supportive treatments were considered appropriate in 95 cases (94.05%) and inappropriate in six cases (5.95%). Specific treatments were judged appropriate in 59 cases (58.41%) and inappropriate in 42 cases (41.58%). These findings underscore the importance of correctly administering treatments - particularly specific antidotes - to improve patient outcomes in poisoning cases (Table 4).

**Table 4:** Details on distribution of poisoning treatment assessment

Assessment of treatment	Number of patients	%
Decontamination method		
Appropriate	98	97.02%
Inappropriate	3	2.98%
Supportive treatment		
Appropriate	95	94.05%
Inappropriate	6	5.95%
Specific treatment		
Appropriate	59	58.41%
Inappropriate	42	41.58%

Our study included a detailed analysis of patient prognosis, survival rates, and cases of mortality. Of the 101 patients included in the study, 83 (82.2%) survived without any significant complications, demonstrating a favourable prognosis. Unfortunately, 12 patients (11.9%) experienced complications during their recovery process, which required extended hospital stays and additional medical interventions. The mortality rate in this cohort was 6.9% (seven patients), with deaths primarily attributed to delays in hospital arrival and the severity of the poisoning agents involved. Notably, early arrival at the hospital (within three hours of exposure) was associated with a significantly higher survival rate (95%) compared to those who arrived later.

## Discussion

The aim of this study was to assess the clinical profiles, treatment modalities, and outcomes among cases of poisoning in an Indian tertiary care hospital. The results of this study can provide useful information on the pattern and efficacy of interventions for poisoning management. In the majority of the poisoning cases (76.9%), pesticides were involved, and among them, the most predominant type was OPC. This kind of observation is not uncommon, even in other studies where excessive use of pesticides has been reported in various agricultural parts of the country, accounting for accidental and intentional poisoning that is often fatal [7]. This substantial proportion of pesticide poisoning incidences implies the immediate necessity for a closer approach to regulation concerning the sale and use of pesticides, in addition to better education regarding safe practices among agricultural workers.

Bite-related poisonings (mainly resulting from snakebites) were another substantial group of cases. This may be why incision, drainage, and other inadequately researched treatments for jejunal ulcers lead to longer recovery times [8]. These findings are consistent with studies from elsewhere in India on another rural health problem (snake bites) due to a large, geographically and anatomically diverse snake population endemic throughout the country [9]. The key to successful management lies in the prompt and proper administration of anti-venom, which was done in the present study, with other supportive treatments kept aside.

Both household and medicinal products played a significant role, albeit in smaller numbers. Cases of rat poison and bleaching powder serve as outstanding examples, highlighting the need for public awareness regarding these components [10]. Furthermore, the rare cases of medicinal poisoning demonstrate the need for more strictly regulated prescriptions and patient compliance education in medicine use. Of the assessment of treatment modalities, decontamination and supportive treatment had high appropriateness, standing at 97.02% and 94.05%, respectively. This implies that the hospital’s initial approach to poison cases has achieved a high degree of correctness [11]. However, 58.41% of the cases were found appropriate for treatments following the cohort definition. This discrepancy suggests that more work is needed to enhance protocols and training for health professionals in the administration of specific antidotes and treatments [12].

In our study, we identified several cases where specific treatments or antidotes were not available for the poisoning agents involved. This was particularly notable for patients who reported to the hospital within six hours of exposure. For instance, certain cases of pesticide poisoning, particularly those involving less common OPCs and other agricultural chemicals, lacked specific antidotal treatments. Similarly, poisonings from certain household chemicals and less common pharmaceutical agents also faced challenges due to the unavailability of specific treatments. Despite the prompt arrival of patients at the hospital, the absence of targeted antidotes necessitated reliance on general supportive care and symptomatic management. This highlights a critical gap in the current medical protocols and underscores the need for expanded access to a wider range of antidotes and specific treatments.

Adequate medical attention was crucial for patients' prognosis. This is a good norm, as according to the literature, more than 70% of patients are expected at the hospital between two and three hours after poison intake, where early intervention can dramatically reduce morbidity and mortality in poisoning cases [13-16]. As delays in hospital arrival were related to the occurrence of complications and their severity, an increase in public awareness concerning signs and symptoms suggestive of poisoning is required to ensure an early hospital visit. This study has drawn attention to the significance of a holistic poisoning management approach that comprises restricting the availability of measures to reduce incidence rates, followed by comprehensive public education and professionals' training.

Upon reviewing our treatment protocols and guidelines, we identified several factors contributing to the observed discrepancies in the administration of treatments and antidotes. Despite the availability of well-established guidelines, some cases did not fully adhere to these protocols. Contributing factors included variations in the clinical experience and training of healthcare providers, the availability of specific antidotes, and the complexity of some poisoning cases that required more nuanced clinical judgment. In some instances, delays in recognizing the specific type of poisoning and initiating the correct treatment also played a role. We have also identified logistical challenges, such as stockouts of certain antidotes and variability in the rapid availability of these agents. To mitigate these issues, we recommend enhanced training programs for healthcare providers focused on the latest poisoning management protocols, regular audits of treatment adherence, and improvements in the supply chain to ensure the availability of necessary antidotes. Addressing these factors can help reduce the margin of error and improve patient outcomes in poisoning cases. Further research and continuous monitoring of treatment practices are essential to ensure adherence to guidelines and optimal care for poisoning patients.

Study limitations show that this study was conducted over a short period of three months, potentially missing seasonal variations in poisoning cases. It was limited to a single tertiary care hospital, which may affect the generalizability of the findings to other regions. Data reliance on patient or caretaker reports may introduce recall bias. Some cases had undetermined intervals between poisoning and hospital arrival, impacting outcome accuracy. Additionally, the study lacked long-term follow-up, limiting insights into prolonged health impacts and recovery. Future studies should consider longer durations, multiple centres, and comprehensive follow-up for more robust data.

## Conclusions

This study summarizes clinical profiles, treatment methods, and outcomes of poisoning cases in an Indian tertiary care hospital, highlighting pesticides, especially OPCs, as a major occupational health risk for agricultural workers. The assessment underscores the need for better decontamination and supportive treatment methods, improved healthcare provider training, and faster initial responses to improve patient outcomes. Timely hospital arrival significantly enhanced outcomes, emphasizing the importance of public awareness about poisoning symptoms that require immediate medical attention. The study advocates for strict pesticide use guidelines, comprehensive chemical education for agricultural workers, and increased public awareness of hazardous household chemicals and medical guidelines to improve poisoning prevention and treatment, ultimately reducing pesticide-related deaths and morbidity in India.
